# Integration of disease-specific single nucleotide polymorphisms, expression quantitative trait loci and coexpression networks reveal novel candidate genes for type 2 diabetes

**DOI:** 10.1007/s00125-012-2568-3

**Published:** 2012-05-16

**Authors:** H. P. Kang, X. Yang, R. Chen, B. Zhang, E. Corona, E. E. Schadt, A. J. Butte

**Affiliations:** 1 Division of Systems Medicine, Department of Pediatrics, Stanford University School of Medicine, 1265 Welch Road, Room X163, Stanford, CA 94305 USA; 2 Lucile Packard Children’s Hospital, Palo Alto, CA USA; 3 Sage Bionetworks, Seattle, WA USA; 4 Department of Genetics and Genome Sciences, Mount Sinai School of Medicine, New York, NY USA

**Keywords:** Genetics of type 2 diabetes, Genomics/proteomics, Mathematical modelling and simulation

## Abstract

**Aims/hypothesis:**

While genome-wide association studies (GWASs) have been successful in identifying novel variants associated with various diseases, it has been much more difficult to determine the biological mechanisms underlying these associations. Expression quantitative trait loci (eQTL) provide another dimension to these data by associating single nucleotide polymorphisms (SNPs) with gene expression. We hypothesised that integrating SNPs known to be associated with type 2 diabetes with eQTLs and coexpression networks would enable the discovery of novel candidate genes for type 2 diabetes.

**Methods:**

We selected 32 SNPs associated with type 2 diabetes in two or more independent GWASs. We used previously described eQTLs mapped from genotype and gene expression data collected from 1,008 morbidly obese patients to find genes with expression associated with these SNPs. We linked these genes to coexpression modules, and ranked the other genes in these modules using an inverse sum score.

**Results:**

We found 62 genes with expression associated with type 2 diabetes SNPs. We validated our method by linking highly ranked genes in the coexpression modules back to SNPs through a combined eQTL dataset. We showed that the eQTLs highlighted by this method are significantly enriched for association with type 2 diabetes in data from the Wellcome Trust Case Control Consortium (WTCCC, *p* = 0.026) and the Gene Environment Association Studies (GENEVA, *p* = 0.042), validating our approach. Many of the highly ranked genes are also involved in the regulation or metabolism of insulin, glucose or lipids.

**Conclusions/interpretation:**

We have devised a novel method, involving the integration of datasets of different modalities, to discover novel candidate genes for type 2 diabetes.

## Introduction

Genome-wide association studies (GWASs) of common complex or multifactorial diseases have proliferated enormously over the last few years. They have also been successful in identifying a large number of loci at extraordinary levels of significance, given the large cohort sizes. However, this success has presented a new challenge: translating these findings into a full understanding of how the loci affect complex disease traits. Most of the reported variants do not affect protein function in an obvious manner and indeed a large number lie in introns or intergenic regions [1], indicating that they may function through other regulatory mechanisms such as control of gene expression or alternative splicing. Even the gene or genes implicated in a given disease with respect to a given genetic locus in such cases is not entirely clear, given that variation near one gene may affect regulation of another neighbouring gene, or variation in the intron of a protein-coding gene may affect the regulation of an embedded non-coding RNA. Variations in DNA affecting the expression of a gene begs the question of whether such genetic variants, commonly known as expression quantitative trait loci (eQTLs), are an important factor in disease susceptibility [2].

The genes regulated by eQTLs are commonly referred to as expression traits. The relationship between eQTL genotypes and expression traits are mapped by performing GWASs for the expression of all genes in the organism of interest [3]. This requires simultaneous measurement of genetic variation (single nucleotide polymorphisms [SNPs]) and gene expression in the same individuals. Multiple hypothesis correction, either through applying a Bonferroni-adjusted cut-off or more sophisticated methods such as permutation-based analysis to empirically estimate the null distribution and control the false discovery rate (FDR), is then used to filter the association results. The SNPs that tag eQTLs are commonly referred to as expression SNPs (eSNPs). However, eQTL-expression trait relationships are not just statistical associations. Nicolae *et al* demonstrated that trait-associated SNPs in general are enriched for eQTLs [4]. In addition, studies have shown that eQTLs mapped in a disease-relevant tissue of interest are enriched for disease-associated SNPs [5, 6]. These insights have been applied to leverage eQTLs in providing a more relevant context within which to interpret SNP associations to disease and to prioritise GWAS results [6–8]. Other investigators have taken advantage of the link between genetic variation and gene expression by integrating eQTLs with coexpression networks and showing that this is a powerful model for discovering novel genes and gene networks relevant to disease [9, 10].

In this study, we demonstrate a novel approach integrating eQTLs and mouse tissue-specific coexpression networks with knowledge of genetic variants reproducibly associated with type 2 diabetes. Our hypothesis was that if the genes coexpressed with expression traits linked to the well-known type 2 diabetes SNPs have any explanatory power or biological relevance for type 2 diabetes, then eSNPs linked with expression traits for these coexpressed genes should already have been associated with type 2 diabetes in previously run case–control GWASs, albeit at a much lower, indirect level of significance. Showing weaker but consistent levels of significance in case–control studies for these other eSNPs would potentially serve as a valuable method for explaining some of the ‘missing heritability’ in the genetic architecture of complex disease, while at the same time serving as a method to explain those variants already discovered in GWASs.

## Methods

**Fig. 1:**
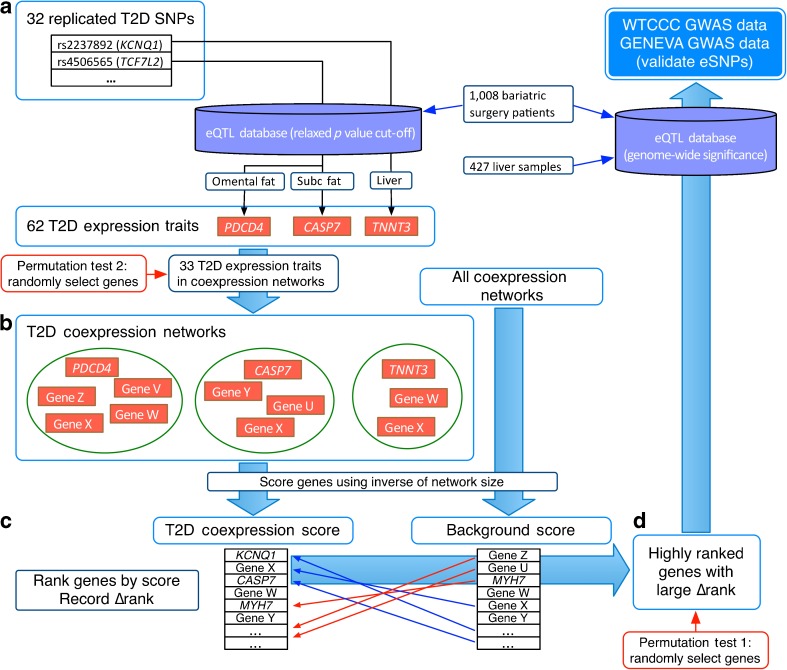
Integrating replicated disease SNPs with differentially expressed genes through an eQTL dataset. (**a**) We mapped 33 SNPs replicated in more than one GWAS for type 2 diabetes to 62 expression traits in liver and omental and subcutaneous adipose tissue taken from morbidly obese individuals. (**b**) There were 526 coexpression networks that contained at least one of these expression traits. (**c**) The score for each gene in the coexpression networks was calculated by taking the sum of the inverse of the network sizes in which it appeared. A background score was also calculated for all coexpression networks, then the two sets of genes were ranked by score and the Δrank was recorded. (**d**) Highly ranked genes with large Δrank were mapped back to eSNPs, and we evaluated the distribution of *p* values of these eSNPs in data from the WTCCC. The two different models for the permutations are also shown. Subc, subcutaneous; T2D, type 2 diabetes

The overall experimental design is shown in Fig. 1. We first constructed a comprehensive dataset of eQTLs discovered in metabolically significant tissues by collecting all SNP–gene expression relationships with genome-wide significance reported in liver and subcutaneous and omental adipose tissue [10, 11].

To identify a starting set of genes associated with type 2 diabetes, we first selected SNPs that had been reproducibly associated with type 2 diabetes in two or more independent GWASs. To map these type-2-diabetes-associated SNPs to candidate causal genes, we used cis SNP-gene associations (gene to SNP distance within 1 Mb) identified with the Kruskal–Wallis test at an uncorrected *p* value cut-off of 0.05 in liver and subcutaneous and omental adipose tissue of 1,008 morbidly obese patients [10]. In total, there were 508 genes within 1 Mb of these SNPs. We decided that the higher false-positive rate resulting from this relaxed cut-off could be tolerated because we planned to use additional filters to genetically validate any novel associations. We refer to these type 2 diabetes candidate genes mapped via eQTLs as type 2 diabetes expression traits.

We obtained previously described tissue-specific coexpression subnetworks constructed from gene expression studies in human tissues [10, 12, 13] and various mouse crosses, including C57BL6/J × C3H/HeJ on both wild type [11] and *Apoe* ^−/−^ backgrounds [9, 14], C57BL6/J × A/J [15] and C57BL6/J × Castaneus [11]. The weighted coexpression subnetworks were constructed with previously described methods using the most highly connected nodes from each tissue [16]. Briefly, the networks are based only on calculated gene–gene pairwise correlations, with the modules in these networks identified based on topological properties in an approach that avoids hard thresholding on the correlations. The weighted network analysis begins with a matrix of the Pearson correlations between all gene pairs and then converts the correlation matrix into an adjacency matrix using a power function *f(x) = x* ^*b*^. The variable *b* of the power function is determined in such a way that the resulting adjacency matrix, namely the weighted coexpression network, is approximately scale-free. By not including literature, canonical pathways and other such relationships among genes, the coexpression networks represent a more unbiased view of the biological processes (reflected in the modules) at play.

Subnetworks that contained at least one type 2 diabetes expression trait were selected; these will be referred to as type 2 diabetes coexpression networks. Genes that appeared in more of these modules could be considered more likely to be associated with type 2 diabetes. We therefore scored genes for type 2 diabetes coexpression by counting the number of modules they appeared in. Because a gene would be more likely to be present in a larger module simply by chance, we weighted the score by taking the inverse of the network size. Therefore, the type 2 diabetes coexpression score for each gene was calculated by summing the inverse of the number of genes in each type 2 diabetes coexpression network containing that gene, or $\sum\nolimits_i^n {1/{S_i}}$, where *n* is the number of networks in which the gene co-occurs with an expression trait and *S*_*i*_ is the number of genes in network *i*. The background score was calculated by repeating this analysis for the genes in all coexpression networks.

Genes were mapped back to eSNPs using the previously mentioned eQTL dataset containing SNP–RNA relationships of genome-wide significance. To test whether the eSNPs of genes above a certain score cut-off were enriched for association to type 2 diabetes, we used SNP summary statistics provided by the Wellcome Trust Case Control Consortium (WTCCC). Because the eQTL mapping studies and the WTCCC assayed SNPs on different platforms with incomplete overlap, the eSNPs were expanded to include those in strong linkage disequilibrium (LD; *R* ^2^ > 0.8). The percentage with *p* < 0.05 for type 2 diabetes (*p*_T2D_ < 0.05) was recorded. Because we would expect type 2 diabetes expression traits and genes containing type 2 diabetes SNPs to score highly using our filters, we removed these from the novel candidate gene lists before performing this evaluation.

We empirically estimated the null distribution of eSNPs using two different models. For the first model, we randomly selected half of the genes above a certain score cut-off, mapped these to eSNPs, expanded into strong LD, and recorded *p*_T2D_ < 0.05 as above. This was repeated 10,000 times for each cut-off. For the second model, we randomly selected 33 genes as the initial expression traits used to determine the subnetworks and repeated the entire analysis. This process was repeated 1,000 times.

We also used an independent GWAS dataset from the Gene-Environment Association Studies (GENEVA) Diabetes Study (release Version 2) to validate our findings. This is a GWAS performed on a total of 3,000 cases and 3,000 controls from two well-characterised cohorts: the Nurses’ Health Study (www.channing.harvard.edu/nhs) and the Health Professionals Follow-up Study (www.hsph.harvard.edu/hpfs). We obtained SNP summary statistics through the Database of Genotypes and Phenotypes (dbGaP; dbGaP Study Accession: phs000091.v2.p1) and performed the same permutation analysis by randomly selecting 33 genes as the initial expression traits.

## Results

### Type 2 diabetes expression traits

We started with 32 SNPs that have been associated with type 2 diabetes in multiple studies (Table 1) [17–22]. Using genotype data of 1,008 patients and gene expression profiles of three tissues (omental adipose, subcutaneous adipose and liver) collected at the time of gastric bypass surgery [10], we identified genes within 1 Mb of each type 2 diabetes SNP with expression correlated with the genotype at that locus. In total, only 21 of these 32 type 2 diabetes SNPs were associated with 62 different expression traits at the uncorrected threshold of *p* = 0.05.

**Table 1:** Thirty-two SNPs reproducibly associated with type 2 diabetes in multiple studies

SNP	Chromosome	Functional type	Adjacent gene
rs7578597^a^	2	Missense	*THADA*
rs4402960^a^	3	Intron	*IGF2BP2*
rs1470579^a^	3	Intron	*IGF2BP2*
rs4607103^a^	3	Intergenic	
rs10010131^a^	4	Intron	*WFS1*
rs7754840	6	Intron	*CDKAL1*
rs7756992	6	Intron	*CDKAL1*
rs10946398	6	Intron	*CDKAL1*
rs4712523	6	Intron	*CDKAL1*
rs864745^a^	7	Intron	*JAZF1*
rs13266634^a^	8	Missense	*SLC30A8*
rs10811661	9	Intergenic	
rs564398^a^	9	Intergenic	
rs7903146^a^	10	Intron	*TCF7L2*
rs12255372^a^	10	Intron	*TCF7L2*
rs7901695^a^	10	Intron	*TCF7L2*
rs11196205^a^	10	Intron	*TCF7L2*
rs7895340^a^	10	Intron	*TCF7L2*
rs4506565^a^	10	Intron	*TCF7L2*
rs1111875^a^	10	Intergenic	*HHEX*
rs5015480^a^	10	Intergenic	*HHEX*
rs12779790^a^	10	Intergenic	
rs5219	11	Missense	*KCNJ11*
rs2237892^a^	11	Intron	*KCNQ1*
rs2237895	11	Intron	*KCNQ1*
rs2237897^a^	11	Intron	*KCNQ1*
rs10830963	11	Intron	*MTNR1B*
rs7961581^a^	12	Intergenic	
rs8050136	16	Intron	*FTO*
rs9939609	16	Intron	*FTO*
rs4430796	17	Intron	*HNF1B*
rs1884613	20	Intergenic	

^a^Serves as an eSNP in liver and adipose tissue

### Scoring genes using mouse coexpression networks

In order to study these 62 expression traits associated with 21 well-known type 2 diabetes SNPs, we screened through 2,326 tissue-specific coexpression modules derived from coexpression networks constructed as previously described [9–16]. Out of the 62 type 2 diabetes expression traits, 33 were present in one or more coexpression network modules, resulting in the implication of 526 type 2 diabetes coexpression network modules.

Each of the 13,961 genes that were present in any of these 526 networks could contribute additional explanatory power as to how those original 21 type 2 diabetes SNPs lead to diabetes. To study each of these 13,961 genes, we calculated the type 2 diabetes coexpression score as $\sum\nolimits_i^n {1/{S_i}}$, where *n* is the number of networks in which the gene is coexpressed with one of the 33 expression traits and *S*_*i*_ is the total number of genes in network *i*. In order to determine a baseline or background score, we treated the entire set of 2,326 coexpression modules as describing all human disease (disease coexpression networks) and calculated the score for all 13,987 genes that appeared in any of these 2,326 coexpression modules. This was done to control for genes present in a high percentage of all networks, for example. We then ranked genes in the type 2 diabetes and background coexpression score lists from highest to lowest scores and recorded the Δrank ([*Background coexpression rank*] − [*Type 2 diabetes coexpression rank*]) for each gene, resulting in a type 2 diabetes coexpression rank and Δrank for each gene. The rank and Δrank for the type 2 diabetes expression traits are listed in Table 2.

**Table 2:** Thirty-three expression traits regulated by type 2 diabetes SNPs

SNP	Adjacent gene	Expression trait	T2D coexpression rank	ΔRank
rs2237892	*KCNQ1*	*KCNQ1*	1	654
rs2237897				
rs2237892	*KCNQ1*	*TNNT3*	3	1,265
rs1111875		*IDE*	23	2,508
rs5015480				
rs1111875		*CYP26A1*	27	2,674
rs5015480				
rs4607103		*ADAMTS9*	34	2,840
rs12255372	*TCF7L2*	*VTI1A*	35	2,843
rs4506565	*TCF7L2*	*CASP7*	42	3,302
rs7895340				
rs11196205				
rs7901695				
rs12255372	*TCF7L2*	*GPAM*	43	3,428
rs5015480		*PDLIM1*	48	3,603
rs1111875				
rs2237897	*KCNQ1*	*TH*	49	3,610
rs7961581		*TSPAN8*	51	3,635
rs864745	*JAZF1*	*TAX1BP1*	101	5,108
rs10010131	*WFS1*	*MRFAP1*	138	5,504
rs564398		*CDKN2A*	145	5,565
rs10010131	*WFS1*	*CPZ*	286	6,449
rs1111875		*TMEM20*	347	6,701
rs5015480				
rs12779790		*DHTKD1*	471	7,012
rs2237897	*KCNQ1*	*SLC22A18*	488	7,047
rs4506565	*TCF7L2*	*DCLRE1A*	748	7,483
rs7903146				
rs7901695				
rs10010131	*WFS1*	*JAKMIP1*	1,288	7,748
rs564398		*PTPLAD2*	1,409	7,774
rs2237892	*KCNQ1*	*CARS*	1,414	7,779
rs2237892	*KCNQ1*	*NAP1L4*	1,955	7,864
rs7961581		*TMEM19*	2,243	7,842
rs4607103		*PSMD6*	2,630	7,790
rs4506565	*TCF7L2*	*PDCD4*	3,496	7,445
rs7901695				
rs7903146				
rs2237892	*KCNQ1*	*MRGPRE*	3,615	7,394
rs13266634	*SLC30A8*	*THRAP6*	4,001	7,211
rs7578597	*THADA*	*ZFP36L2*	4,266	7,117
rs12255372	*TCF7L2*	*RBM20*	4,870	6,788
rs864745	*JAZF1*	*HOXA13*	5,330	6,535
rs10010131	*WFS1*	*STK32B*	5,457	6,470
rs12779790		*SEC61A2*	7,895	4,828

ΔRank, the difference between type 2 diabetes coexpression rank and background rank; T2D coexpression rank, the rank of each gene’s type 2 diabetes coexpression score

### eSNPs of highly ranked genes are enriched for association with type 2 diabetes

As stated above, our hypothesis was that if the genes coexpressed with expression traits linked to the well-known type 2 diabetes SNPs have any explanatory power or biological relevance for type 2 diabetes, then variants in these genes might have already been associated with type 2 diabetes in case–control studies, albeit at a much lower, indirect, level of significance. We thus evaluated the performance of the type 2 diabetes coexpression score and rank change for selecting genes relevant to the pathogenesis of type 2 diabetes. Of the 13,961 genes coexpressed with the expression traits of type 2 diabetes SNPs, 6,030 (6,005 excluding type 2 diabetes expression traits) could be mapped back through eQTLs to 20,480 SNPs that had been tested in the well-known GWAS on type 2 diabetes, run by the WTCCC.

**Fig. 2:**
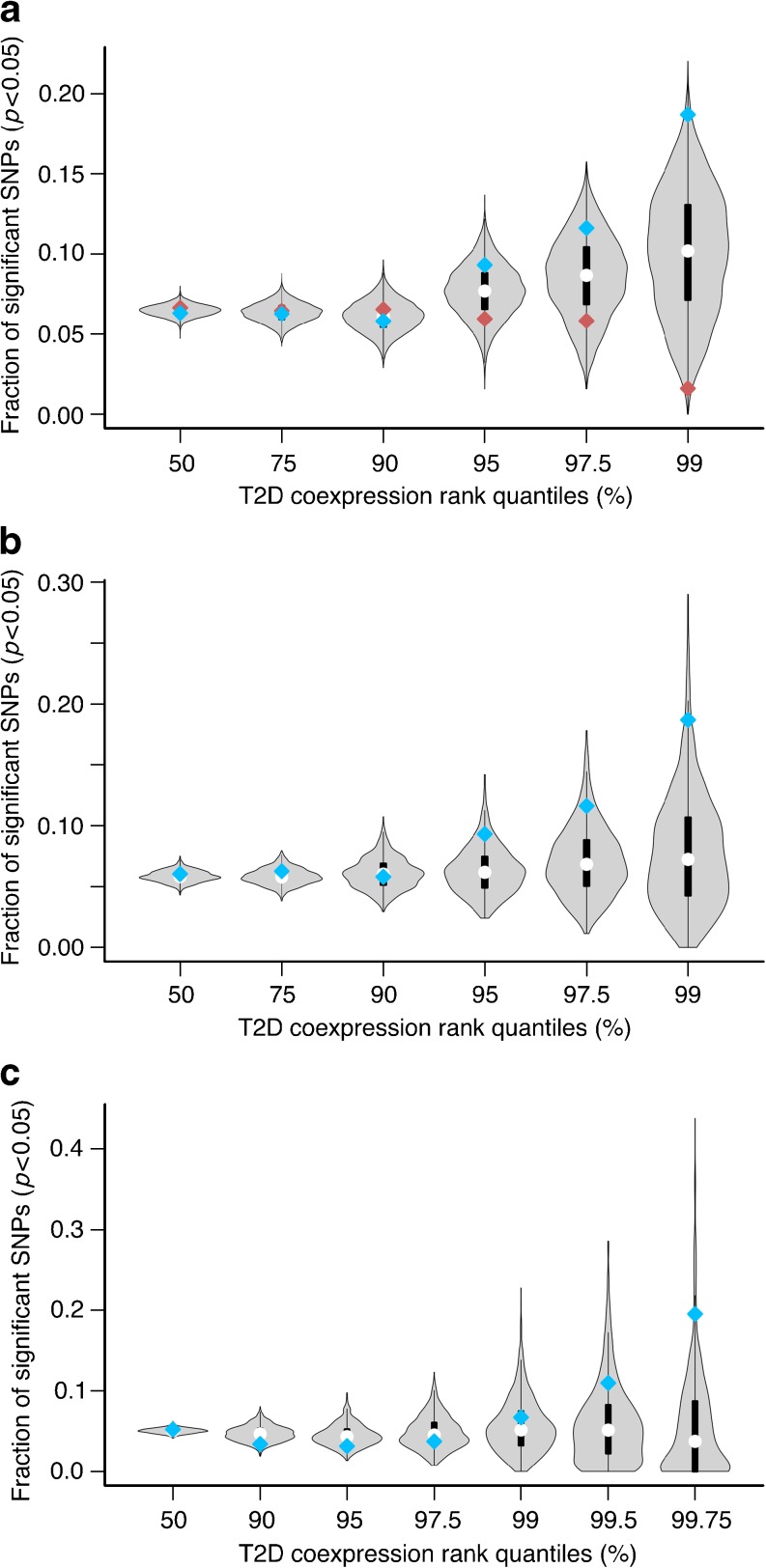
(**a**) Enrichment of *p*_T2D_ < 0.05 at increasing type 2 diabetes coexpression score quantiles. The genes in each quantile were ordered by decreasing Δrank, then split at the median Δrank. The genes in each half quantile were connected to eSNPs using eQTL datasets mapped in liver, omental and subcutaneous adipose tissue. The blue and red points represent, respectively, the *p*_T2D_ < 0.05 for the top and bottom halves. To estimate the null distribution, we randomly selected half of the genes in each group 10,000 times and observed the *p*_T2D_ < 0.05 for each sample. The violin plots represent the distribution of the permutations for each set of genes. The separation between the top and bottom halves as the threshold is increased suggests that the coexpression rank, coupled with the Δrank, is a useful metric for selecting genes relevant to the disease process. *p* = 0.12 for 97.5 percentile; *p* = 0.007 for 99 percentile. (**b**) Estimation of the null distribution by permuting the initial expression traits. As another estimate of the null distribution, we randomly selected 33 genes as the initial expression traits. As in (**a**), we then observed the *p*_T2D_ < 0.05 in WTCCC data for the top half of genes at each cut-off for each sample. The violin plots represent the distribution of this value at each cut-off for each sample. We repeated this permutation analysis 1,000 times. *p* = 0.066 for 97.5 percentile; *p* = 0.026 for 99 percentile. (**c**) Validation of results using an independent dataset. To validate our findings shown in (**b**), we repeated the permutation analysis using data obtained from the GENEVA diabetes study. *p* = 0.13 for 99.5 percentile; *p* = 0.042 for 99.75 percentile. Blue diamond, *p*_T2D_ < 0.05 for genes with change in rank greater than quantile median; red diamond, *p*_T2D_ < 0.05 for genes with change in rank less than quantile median. T2D, type 2 diabetes

We selected sets of these 6,005 genes at six type 2 diabetes coexpression rank quantile cut-offs (0, 75, 90, 95, 97.5, 99). Although there was a trend for increasing enrichment of WTCCC *p*_T2D_ < 0.05 in the higher quantiles, it was not significant. Because we expected genes relevant to type 2 diabetes to have a high Δrank compared with background, we ordered each quantile by Δrank, then split each set into those above and below the median Δrank for that quantile. We compared the WTCCC *p*_T2D_ < 0.05 of the subsets of genes above and below the median Δrank (Fig. 2a), revealing the genes with high Δrank in each set to be increasingly enriched for WTCCC *p*_T2D_ < 0.05 at higher cut-offs and thus confirming our original hypothesis.

The null distributions for the halves of each quantile were estimated by randomly selecting half of the genes in each set and recording the *p*_T2D_ < 0.05. This was repeated 10,000 times. This analysis revealed that the enrichment trend observed in Fig. 2a reached significance at the 99th percentile cut-off (18.7%, *p* = 0.007). We also performed a more stringent estimation of the null distribution by randomly selecting 33 genes as the initial expression traits and replicating the analysis 1,000 times. The result of permutation using this model was consistent with the first one, with a *p* value of 0.026 at the 99th percentile cut-off (Fig. 2b). In order to validate these findings, we repeated the analysis using SNP data from the GENEVA diabetes study. A total of 7,047 genes (7,037 excluding type 2 diabetes expression traits) could be mapped back through eQTLs to 31,011 SNPs tested in this study. Similar to what we had seen with WTCCC, we observed significant enrichment (19.5% in the 99.75th percentile, *p* = 0.042) for GENEVA *p*_T2D_ < 0.05 at higher cut-offs.

The genes in the 99th percentile cut-off that mapped to WTCCC are listed in Table 3. Eight of these genes have eSNPs with *p* < 0.05 for type 2 diabetes. Two (*HMGCS1*, *IDI1*) are involved in steroid synthesis and one (*KHK*) is involved in monosaccharide metabolism.

**Table 3:** Top 30 candidate type 2 diabetes genes, out of 6,005, with the selected type 2 diabetes expression traits with which they are coexpressed

Gene containing T2D SNP	Expression trait	Coexpressed candidate gene	Coexpression rank	ΔRank
*TCF7L2*	*VTI1A*	*TNNC1*	14	1,492
*TCF7L2*	*CASP7*	*KBTBD10*	19	1,886
*TCF7L2*	*DCLRE1A*	*PGAM2*	21	1,960
*KCNQ1*	*TNNT3*	*CKMT2*	32	2,020
*WFS1*	*MRFAP1*	*HRC*	37	1,457
*KCNQ1*	*NAP1L4*	*TXLNB*	38	1,230
*SLC30A8*	*THRAP6*	*TCAP* ^a^	54	2,668
*WFS1*	*CPZ*	*RYR1*	55	1,241
*THADA*	*ZFP36L2*	*FRY* ^a^	60	2,624
*KCNQ1*	*KCNQ1*	*LMOD2*	63	1,465
*KCNQ1*	*CARS*	*CACNA1S*	64	3,171
*JAZF1*	*TAX1BP1*	*ECHDC1*	65	3,189
*TCF7L2*	*PDCD4*	*NAV2*	66	2,535
*WFS1*	*STK32B*	*TMPRSS4*	67	1,798
*KCNQ1*	*TH*	*MLF1*	72	1,628
Intergenic	*CDKN2A*	*KIAA0323* ^b^	78	1,850
*KCNQ1*	*SLC22A18*	*ANKRD33*	87	3,086
Intergenic	*TSPAN8*	*PYGM*	88	2,481
*KCNQ1*	*MRGPRE*	*IDI1* ^a^	89	1,220
Intergenic	*TMEM20*	*MTHFD1L*	97	2,701
Intergenic	*SEC61A2*	*HMGCS1* ^a^	98	1,927
Intergenic	*PSMD6*	*CD44* ^a^	106	1,321
*TCF7L2*	*GPAM*	*LMCD1*	107	2,950
*WFS1*	*JAKMIP1*	*PTGDR* ^a^	109	4,974
*JAZF1*	*HOXA13*	*FADS2*	114	1,529
Intergenic	*ADAMTS9*	*MYOM1* ^a^	117	1,754
Intergenic	*IDE*	*PDLIM3*	119	2,177
*TCF7L2*	*CASP7*	*INSIG1*	120	1,529
*KCNQ1*	*KCNQ1*	*PFKM*	121	2,902
Intergenic	*PTPLAD2*	*KHK* ^a^	124	2,722

Coexpression rank, the rank of the type 2 diabetes coexpression score for each gene ΔRank, the difference between type 2 diabetes coexpression rank and background rank

^a^Any linked eSNP with *p* < 0.05 in WTCCC

^b^Also known as *KHNYN*

T2D, type 2 diabetes

## Discussion

GWASs are revealing increasing numbers of loci associated with various diseases. However, our understanding of the biological mechanisms behind these genetic variants is, in many cases, incomplete. eQTLs have the potential to aid in deciphering these variants by associating them with gene expression.

Although a recent study discovered no significant eQTL relationships for well-known type 2 diabetes SNPs in colon, pancreas or liver tissue [23], our initial analysis of type 2 diabetes expression traits revealed several such examples. This most likely reflects the large number of individuals from whom we could collect samples, as well as our inclusion of adipose tissue in this study. For instance, the expression of *TCF7L2* is not associated with any of the SNPs studied, but there are several genes, including *VTI1A*, *PDCD4* and *CASP7*, whose expression is associated with SNPs in *TCF7L2*. *VTI1A*, which we observed as an expression trait of the *TCF7L2* SNP in omental adipose tissue, is a vesicle-soluble NSF attachment protein receptor (v-SNARE) that is a component of insulin-sensitive *GLUT4*-containing vesicles and affects insulin-dependent glucose transport in adipocytes [24]; *PDCD4*, another expression trait of the same SNP, plays a crucial role in pancreatic beta cell death in type 1 diabetes [25]; *CASP7* has also been identified as a positional candidate gene for type 1 diabetes [26]. All of these expression traits could serve as causal explanations of how the *TCF7L2* SNP leads to type 2 diabetes.

As another example, rs1111875 and rs5015480, variants located close to the *HHEX* gene, are actually associated with the expression of *IDE* (encoding insulin-degrading enzyme) in subcutaneous fat. This association suggests that the functional significance of these SNPs in type 2 diabetes is relayed through the expression of *IDE*, which plays a central role in insulin metabolism [27]. In other words, explaining GWAS findings using eSNPs, as demonstrated here, might help to distinguish between two nearby genes with radically different potential mechanisms for disease.

Finally, rs564398, a variant in *CDKN2B* antisense RNA 1 (*CDKN2B-AS1*), is associated with the expression of *CDKN2A* in omental adipose tissue and *PTPLAD2* in liver. A recent study reported that this SNP was associated with the expression of *CDKN2B-AS1* but not *CDKN2A/B* in peripheral blood [28]; our result may be specific to the tissue types that we investigated. Here, our analysis using eSNPs suggests several different candidate mechanisms across separate tissues; it could be that higher-significance variants for type 2 diabetes play their role through distinct mechanisms in multiple relevant tissues.

In this study, we devised a ranking system that uses these type 2 diabetes expression traits in combination with coexpression networks in metabolically important tissues to discover novel genes associated with type 2 diabetes. By combining eQTL datasets from two different studies, we discovered that eSNPs regulating highly ranked genes in these tissues had a significant rising trend for association with type 2 diabetes in two well-known GWASs, specifically those performed by the WTCCC and GENEVA initiative. While the trend observed in the GENEVA study reached significance at a higher quantile cut-off, we think this is still sufficient to confirm our findings.

Having thus confirmed our hypothesis, we reasoned that the highly ranked genes that were not even marginally associated with type 2 diabetes in a GWAS might also be relevant to the pathogenesis of type 2 diabetes. We therefore investigated the other novel genes highlighted by our algorithm. Many of these novel genes that we identified are primarily expressed in skeletal or cardiac muscle. Several of these are involved in insulin signalling or glucose metabolism. For example, Ca^2+^ influx through L-type Ca^2+^ channels (*CACNA1S*) is essential for glucose-stimulated insulin secretion [29], while sarcosin (*KBTBD10*) is a cytoskeletal protein that, like *VTI1A*, is associated with the insulin-stimulated glucose transporter *GLUT4*; interestingly, this association is suppressed in the presence of insulin [30]. *PYGM* (phosphorylase, glycogen, muscle) and *PFKM* (phosphofructokinase, muscle) are key enzymes in glycogenolysis and glycolysis.

We also discovered several genes involved in cholesterol (*INSIG1*, insulin induced gene 1; *HMGCS1*, HMG-CoA synthase 1; *IDI1*, isopentenyl diphosphate isomerase 1) and fatty acid (*FADS2*, fatty acid desaturase 2; *ECHDC1*, enoyl CoA hydratase domain containing 1) metabolism. In particular, *FADS2* activity has been linked to the risk of developing type 2 diabetes [31].

Fructokinase (*KHK*) is another interesting candidate gene revealed by our analysis. The endproduct of *KHK* is fructose-1-phosphate, which accelerates release of glucokinase (*GK*) from its regulatory protein (*GKRP*) [32]. Glucokinase serves as an insulin sensor in the pancreatic beta cells and is being evaluated as a potential drug target for type 2 diabetes [33].

The method presented here represents a paradigm for using eQTLs and prior knowledge of SNPs associated with a disease to discover additional candidate genes and variants for that disease. The strength of this approach lies in the fact that it incorporates the functional significance of the SNPs encapsulated in the association of eQTLs and expression traits. In addition, using coexpression networks constructed in various tissues enables the discovery of candidate genes not expressed in the tissues used for mapping eQTLs. As the number and quality of tissue-specific eQTL studies increase and improve, we anticipate that the power of this type of analysis to detect novel associations will also be enhanced dramatically. This highlights once again the importance of making this type of data available, so that the greater community of scientists may benefit.

## Abbreviations

eQTL: Expression quantitative trait locus
eSNP: Expression single nucleotide polymorphism
GENEVA: Gene-Environment Association Studies
GWAS: Genome-wide association study
LD: Linkage disequilibrium
SNP: Single nucleotide polymorphism
WTCCC: Wellcome Trust Case Control Consortium
